# Supplementary material for: Evaluation of a Frozen Micro-Agar Plates of MAPt Antibiotic Susceptibility Test for Enhanced Bioterror Preparedness
Source: Antibiotics (Basel). 2022 Apr 26;11(5):580. doi: 10.3390/antibiotics11050580 (PMC9137970; doi:10.3390/antibiotics11050580)
Supplement: Supplementary file 1 [file antibiotics-11-00580-s001.zip › antibiotics-1691834-supplementary.pdf]

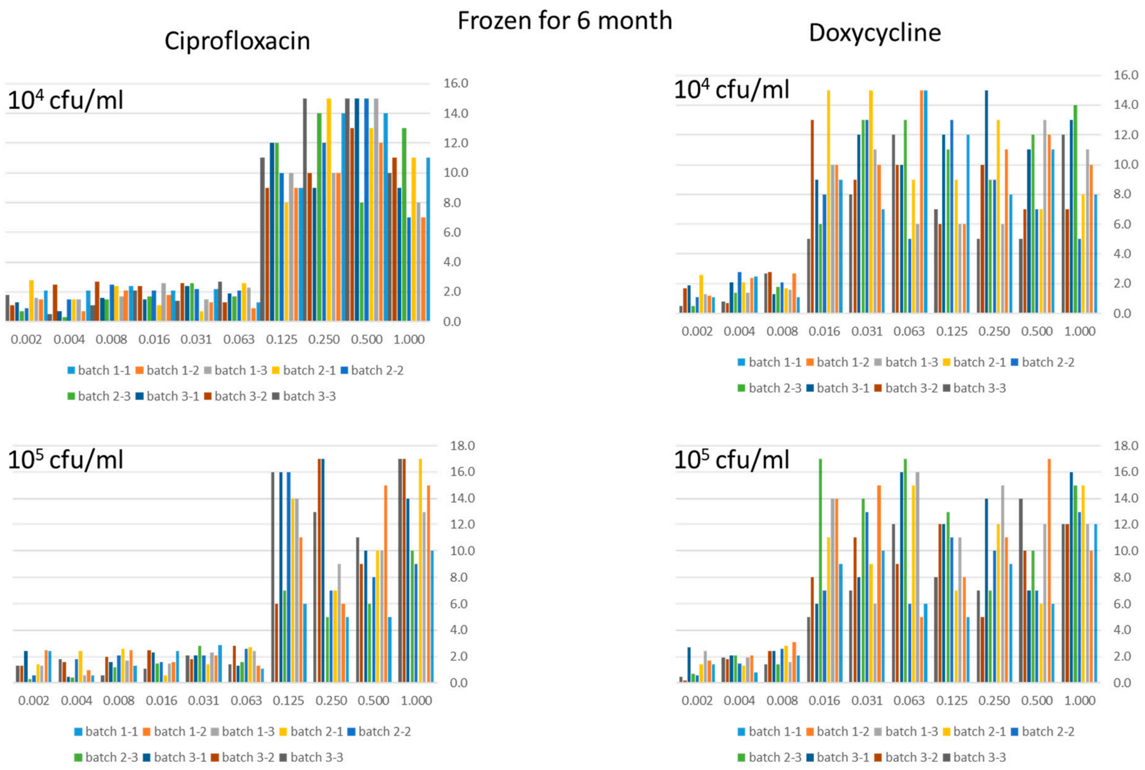

Figure S1. Micro-Agar-PCR-test (MAPt) for 6 month frozen plates. Cultures of *Bacillus anthracis* at 10^4 or 10^5 cfu/ml, were subjected to MAPt testing on ciprofloxacin and doxycycline. The *B. anthracis* plates were incubated for 6 h at 37 °C. Following incubation the bacteria were harvested from the MAPt plate and subjected to qPCR quantification. Minimal inhibitory concentration (MIC) was determined, as described in materials and methods, by the thresholds of $\Delta CT = 3.3$.

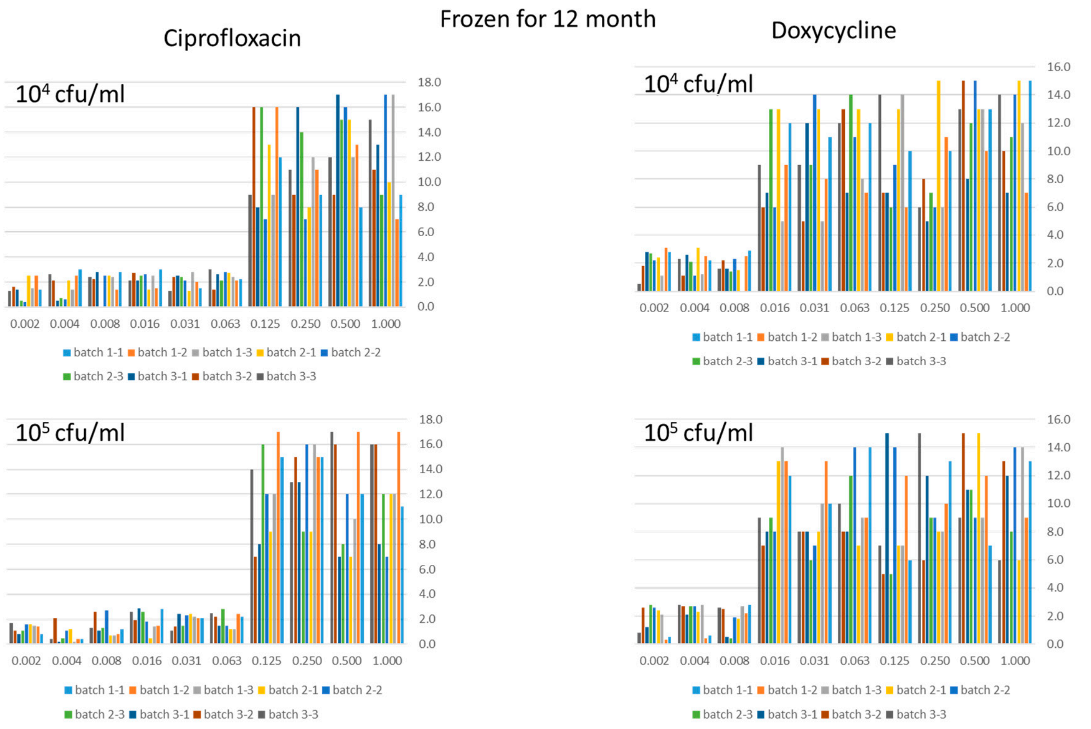

Figure S2. Micro-Agar-PCR-test (MAPt) for 12 month frozen plates. Cultures of *Bacillus anthracis* at 10^4 or 10^5 cfu/ml, were subjected to MAPt testing on ciprofloxacin and doxycycline. The *B. anthracis* plates were incubated for 6 h at 37 °C. Following incubation the bacteria were harvested from the MAPt plate and subjected to qPCR quantification. Minimal inhibitory concentration (MIC) was determined, as described in materials and methods, by the thresholds of ΔCT = 3.3.

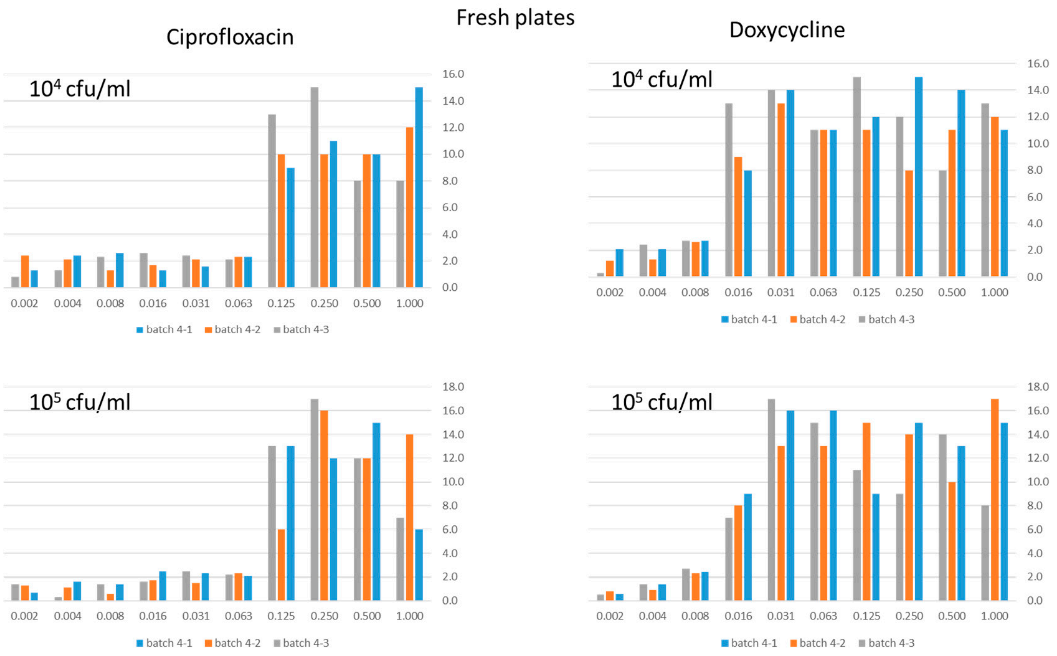

Figure S3. Micro-Agar-PCR-test (MAPt) for freshly prepared plates. Cultures of *Bacillus anthracis* at 10^4 or 10^5 cfu/ml, were subjected to MAPt testing on ciprofloxacin and doxycycline. The *B. anthracis* plates were incubated for 6 h at 37 °C. Following incubation the bacteria were harvested from the MAPt plate and subjected to qPCR quantification. Minimal inhibitory concentration (MIC) was determined, as described in materials and methods, by the thresholds of ΔCT = 3.3.
